# Evaluation of Serum Volume Losses During Long-Term Storage

**DOI:** 10.6028/jres.098.027

**Published:** 1993

**Authors:** Neal E. Craft, Katherine S. Epler, Therese A. Butler, Willie E. May, Regina G. Ziegler

**Affiliations:** National Institute of Standards and Technology, Gaithersburg, MD 20899-0001

**Keywords:** density, long-term storage, serum, sodium, sublimation, volume loss

## Abstract

Aliquots of serum collected in a large case-control study of cervical cancer were stored at −70°C for up to 4 years during implementation of the study. When 500 μL serum aliquots were thawed in preparation for carotenoid and vitamin A assays, volumes were noticeably variable and fell below 500 μL in the majority of the samples. We were concerned about evaporation/sublimation during storage of the samples because loss of water would concentrate the analytes of interest. We evaluated the use of density and sodium ion concentration measurements to confirm its occurrence. We found that serum density was an unreliable indicator of extent of volume loss since the anticipated increases in density due to evaporation were of the same magnitude as inter-individual variation in serum density. In contrast, Na^+^ concentration is tightly regulated and would rise if water had been lost from the samples. In a representative sample of serum aliquots from the case-control study, 24 of 25 vials contained less than 500 μL of serum. The mean sodium ion concentration (138.1 ± 3.6 mmol/L) was within the normal range for human serum of 136–145 mmol/L, and no correlation was observed between serum volume and Na^+^ concentration. These results strongly suggest that the observed low volumes were not due to evaporative losses. Instead, the variably low volumes of serum aliquots were probably due to pipetting errors in the initial aliquotting resulting from the use of air-displacement pipettes.

## 1. Introduction

To study the determinants of disease in human populations, epidemiologists are increasingly relying on biochemical measurements in human tissues, such as blood, to complement information obtained by interviews. “Molecular epidemiology” requires reliable methods for sampling and storing biological materials collected from large numbers of individuals in the field. For example, in a prospective epidemiologic study, blood may be collected from a healthy group of people, and the cohort followed over time. When a sufficient number of diagnoses or deaths have occurred in the cohort, which may require many years, the blood samples collected earlier can be compared for the cases and a subset of the non-cases matched to the cases. Aliquots of the stored blood are thawed and assayed for molecules of interest, such as nutrients, hormones, viral markers, putative carcinogens, etc. Alternatively, in a retrospective epidemiologic study, patients with a particular disease are identified, comparable controls are selected, and blood samples can be collected and compared for the cases and controls. Aliquots of the blood often need to be stored for several years until sufficient numbers of subjects are identified so that assays can be run consecutively on all samples with tight quality control. In both types of epidemiologic studies, blood is often collected under non-optimal conditions, such as at the home, workplace, or neighborhood clinic, to maximize subject participation.

In a large case-control study of cervical cancer conducted in five areas of the United States [1,2], blood to be assayed for micronutrients believed to be involved in the etiology of this cancer was collected from 1023 subjects. Blood was drawn at least 6 mo after completion of surgery or other treatment for cervical cancer to allow dietary patterns, appetite, and metabolism to revert to normal. Aliquots of serum were prepared, mixed with any reagents necessary to stabilize analytes of interest, and stored at −70 °C for up to 4 yr while the study was being conducted. When 500 μL serum aliquots were defrosted in preparation for carotenoid and vitamin A assays, volumes were noticeably variable and tended to be less' than 500 μL in the majority of the samples. We were concerned about evaporation/sublimation during storage because a loss of water would have concentrated the analytes. Other workers have reported losses from polypropylene bottles to be 0.26% [3] and 0.27% [4] per year at room temperature. This low percentage can be of significance when very small initial sample volumes are involved. Such samples would have a much higher relative surface area than larger volumes would have. Another group of workers has discussed evaporative losses from open autosampler cups, and has expressed surprise that the clinical literature has failed to discuss this problem fully [5]. Here we describe the use of serum density and sodium ion (Na^+^) concentration to determine whether evaporative volume loss occurred from polypropylene cryovials during long-term storage at −70°C.

## 2. Materials and Methods

### 2.1 Specimens

Twenty-five of the samples were 500 μL aliquots of serum from subjects in a case-control study of cervical cancer (study samples). Blood had been collected from the participating subjects by a single trained phlebotomist. After centrifugation serum was aliquoted with a 0.1 to 1.0 mL adjustable-volume, air-displacement pipet into 1.2 mL polypropylene cryovials with inside-thread screw stoppers and silicone gaskets. The samples had been stored in a −70°C frost-free freezer for 4–5 yr prior to this analysis. In addition, ten samples were tested from each of two serum pools (QCl and QC2), prepared with known amounts of specific micronutrients to monitor quality control. Approximately 500 μL of each of the QC pools had been aliquoted with an automatic dispenser into similar 1.2 mL cryovials and stored at −70 °C for 4 yr prior to this analysis. One individual aliquotted the study samples; a second individual aliquotted the QC samples. The QC samples were included in this volume-loss study because each set came from one serum pool. Therefore, the parameter being measured, either density or Na^+^ concentration, should have been the same for each of the ten samples in the set if no evaporation or sublimation had occurred. If, however, such a loss had occurred, the differences between results for these samples would have been noticeable since the volumes of serum in these cryovials were also variable, suggesting that if evaporation/sublimation had indeed occurred, it had not been at the same rate for all cryovials.

### 2.2 Density

Serum samples were thawed at room temperature with screw caps on and were then centrifuged at 1000 ×*g* for 5 min to force condensation on the cap down to the bottom of the tube. Sample volumes were measured to ±1% using a calibrated 500 μL gas-tight syringe. Each sample was weighed to ±2 mg using an electronic balance. The density of each serum sample was calculated by dividing the weight by the volume.

### 2.3 Sodium Ion Concentration

Na^+^ concentration was determined by flame atomic emission spectroscopy (FAES) using a modification of the NIST reference method for the determination of sodium in serum [6]. Briefly, the samples were gently mixed, and a 200 μL aliquot of each sample was diluted 1:100 with KCl diluent (4.5 mmol/L). Following calibration and method verification using NIST SRM 909: Freeze-dried Human Serum, Na^+^ was measured by FAES at 589 nm. The precision of the method is estimated at ± 1% with a bias of less than 1.5%.

### 2.4 Statistical Analysis

Linear regression analysis was performed with the data from the cervical cancer samples to determine the correlation coefficients (*r*) for density vs volume and Na^+^ concentration vs volume. Differences in volume, density, and Na^+^ concentration among the three groups of samples were determined by analysis of variance using General Linear Models^2^ (SAS, Cary, NC) [7].

## 3. Results

### 3.1 Serum Volume and Density

**Fig. 1:**
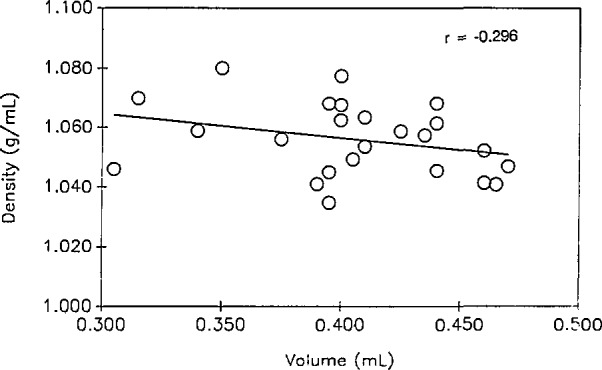
Scatterplot of density and volume for 25 serum samples stored at −70 °C for 4–5 yr, from participants in a case-control study of cervical cancer.

The volume of 24 of the 25 study samples was less than 500 μL, ranging from 305 to 470 μL. This range as well as the mean density, Na^+^ range, and mean Na^+^ concentrations are provided in Table 1. Twenty-five percent of the samples contained less than 406 μL; 50%, less than 430 μL; and 75%, less than 465 μL. By contrast, the volume of the 10 QC1 samples ranged from 495 to 515 μL; and the 10 QC2 samples ranged from 410 to 440 μL. The mean volumes were significantly different (*p* < 0.001) among the three groups. The correlation coefficient between density and volume was −0.296 for the study samples (Fig. 1). QC sera were not included in the linear regression analysis since they are aliquots of the same sera, and the QC sera were not necessarily representative of normal sera.

### 3.2 Sodium Ion Concentration

**Fig. 2:**
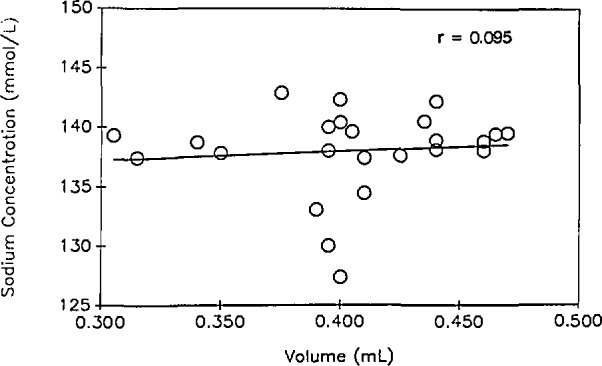
Scatterplot of Na^+^ concentration and volume for the same 25 samples described in Fig. 1.

The Na^+^ concentrations for the 10 QC1 samples were essentially the same (146.6 ±1.3 mmol/L); likewise the QC2 samples were not different from each other (140.4 ±1.3 mmol/L). The mean of the QC2 group was within the normal range of human serum Na^+^ concentrations of 136–145 mmol/L [8]. The mean Na^+^ concentration of QC1 was approximately 1% above the normal range. The range of Na^+^ concentrations in the samples from the 25 case-control study subjects was wider (127.4–142.9 mmol/L) than the range for either QC series; the mean concentration (138.1 ±3.6 mmol/L) was within the normal range. As illustrated in Fig. 2, no correlation (*r* = 0.095) was found between Na^+^ concentration and volume in the study samples.

## 4. Discussion

We investigated the cause of apparent volume losses during long-term serum sample storage at −70 °C by measuring density and Na^+^ concentration. In preliminary studies of the density of lyophilized sera reconstituted with graded volumes of water, we were able to establish a linear relationship between density and volume over the range of 60 to 100% of the original serum volume. However, density is not a very sensitive indicator of volume loss in serum since the concentration of serum solutes is low and thus the density of serum does not differ greatly from the water that may be evaporating from it. A 20% change in serum volume was required to produce a 1% change in density. The density of normal serum varies by 1–2% [8], which makes density an unreliable measure of evaporative volume loss.

The density of the study samples varied from 1.035 to 1.078 g/mL. This ±2% range in density would be equivalent to ±40% differences in volume, (i.e., 4% change in density = 80% change in volume). There was a slight inverse correlation *(r = −*0.296) between density and volume in the study samples, which might indicate minor evaporation or sublimation losses. The occurrence of serum volume losses during storage of samples is a plausible explanation for the increases in measured concentrations of serum retinol reported by Parkinson and Gal [9].

Serum Na^+^ concentration is very tightly regulated [8,10]. Since Na^+^ concentration varies inversely and proportionately with volume loss, the measurement of Na^+^ concentration could provide a sensitive means of determining whether consistently low serum volumes might be due to actual volume loss during storage or to pipetting errors. Due to the stability of inorganic ions, this approach also offered a possible means of correcting for volume losses in stored serum samples if such losses occurred. The absence of correlation between Na^+^ concentration and volume (Fig. 2) does not support the hypothesis that evaporation or sublimation caused the reduced sample volumes. Among the study samples, only four had Na^+^ concentrations outside the normal range. The Na^+^ concentrations for all four study samples were lower than the norm, again refuting the possibility that evaporative losses might be responsible for low sample volumes. Thus, pipetting errors were probably responsible for the range of volumes observed.

The mean volume of the QC1 samples was not significantly less than the expected 500 μL, and the mean Na^+^ concentration was only slightly above the normal range indicating that volume losses had not occurred in this group of sera. The mean volume of the QC2 samples was significantly less than the expected 500 μL, but the volumes fell within a narrow range. In spite of the low volumes, the mean Na^+^ concentration of these sera was also normal. Had the 17% reduction in volume been due to evaporative losses, the Na^+^ concentration would have been elevated by a corresponding 17%. In another set of QC2 samples used in a preliminary study the Na^+^ concentration was similar and the mean volume was very close to the expected 500 μL. These results may indicate that the dispensing pipette had been incorrectly calibrated for a portion of the QC2 samples.

This exercise indicates that the variably low volumes observed in this study were not due to sample evaporation or sublimation during storage. It also indicates that accurate dispensing, assumed to occur in such studies, may not take place. In some studies aliquotting may not be crucial, e.g., when an accurately measured aliquot is subsequently removed and used for analysis. However, in other studies, dispensing of accurate volumes is critical. Such is the case when an aliquot of serum is added to a measured volume of a solution designed to stabilize the analyte of interest, e.g., the addition of metaphosphoric acid to sera to be analyzed for vitamin C. Unless a tightly regulated serum component, such as Na^+^, is measured to normalize the measurements, a sizeable error could occur. This potential source of error could be greatly reduced if positive-displacement pipettes were used to aliquot viscous fluids such as serum. Air-displacement pipettes are designed for accurate and precise measurement of solutions with a density and viscosity similar to water. However, with viscous fluids, air-displacement pipettes fill and expel incompletely. Errors resulting from incomplete expulsion can be minimized by reversed-mode pipetting, but this is not as convenient as using a positive-displacement pipette. The piston in a positive-displacement pipette comes into contact with the sample, wiping the inside of the plastic pipette tip and making expulsion complete. Because of this contact, the piston must be rinsed or replaced to eliminate carryover errors. Variation in these mass-produced pistons causes the positive-displacement pipette to be inherently less precise than air-displacement pipettes, because the piston in the air-displacement pipette is permanent. Nevertheless the positive-displacement pipette is more accurate and precise than the air-displacement pipette when measuring viscous solutions

In summary, using serum Na^+^ concentrations, we have established that variable low volumes observed during long-term sample storage were not due to evaporation or sublimation but were more likely due to pipetting errors that occurred during the initial sample preparation.

## Figures and Tables

**Table 1:** Volumes actually present in the cryovials, mean density, and the mean and range of the Na^+^ concentration

	Density (g/mL)	Volume (μL)	Na^+^ Range (mmol/L)	Na^+^ (mmol/L)
Study samples	1.056 ±0.012	305 to 470	127.4–142.9	138.1 ± 3.6
QC1	1.051 ± 0.007	495 to 515	144.6–147.7	146.6 ±1.3
QC2	1.052 ± 0.013	410 to 440	138.9–142.8	140.4±1.3
Expected		500	136–145	

## Footnotes

2 Certain commercial equipment, instruments, or materials are identified in this paper to specify adequately the experimental procedure. Such identification does not imply recommendation or endorsement by the National Institute of Standards and Technology, nor does it imply that the materials or equipment identified are necessarily the best available for the purpose.
